# Protocol for 3D direct reprogramming of human glia into dopaminergic neurons with gene expression and immunocytochemistry validation

**DOI:** 10.1016/j.xpro.2025.104088

**Published:** 2025-09-12

**Authors:** Jessica Giacomoni, Kerstin Laurin, Malin Parmar, Mette Habekost

**Affiliations:** 1 Developmental and Regenerative Neurobiology, Wallenberg Neuroscience Center, Lund Stem Cell Center, Department of Experimental Medical Science, Lund University, 221 84 Lund, Sweden

**Keywords:** Cell Biology, Cell culture, Genomics, RNA-seq, Molecular Biology, Gene Expression, Neuroscience, Stem Cells, Cell Differentiation

## Abstract

Direct reprogramming of somatic cells into neurons offers a promising strategy for studying neurological disorders and developing cell-based therapies. This protocol describes the 3D direct reprogramming of human glial progenitor cells into dopaminergic neurons. It includes spheroid formation, lentiviral transduction, neuronal induction using doxycycline, and validation via gene expression and immunocytochemistry. This versatile approach enables efficient reprogramming of human cells into dopaminergic neurons.

For complete details on the use and execution of this protocol, please refer to Giacomoni et al.^1^

## Highlights

• Steps for thawing and expanding human glial progenitor cells before reprogramming
• Instructions for co-seeding glial cells with lentivirus to initiate 3D spheroid formation
• Guidance on applying media changes to transduced spheroids to induce neuronal conversion
• Procedures for validating neuronal identity using gene expression and immunostaining

## Abstract (editor highlights)

Publisher’s note: Undertaking any experimental protocol requires adherence to local institutional guidelines for laboratory safety and ethics.

## Graphical abstract

**Figure:**
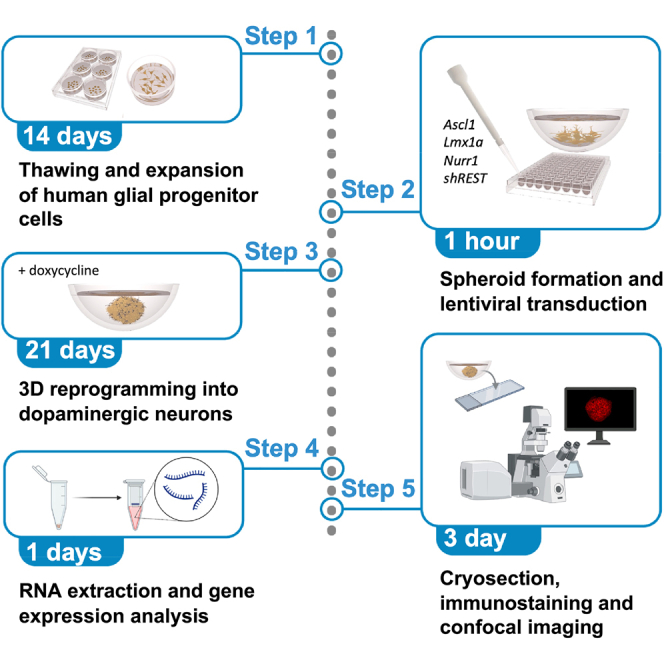

## Before you begin

This protocol outlines spheroid formation, lentiviral transduction, neuronal induction, and validation via gene expression analysis and immunocytochemistry. Before starting, prepare tissue culture plates as instructed, aliquot reagents and small molecules, and prepare culture media following the provided recipes. Lentiviral vectors must be produced and titrated (detailed instructions are available in^2^ with plasmids accessible from public repositories). Cell stocks, such as cryopreserved hGPCs, must be acquired or produced and validated. Protocols for glial differentiation and cryopreservation procedures can be found in.^3^

### Innovation

The ability to generate subtype-specific neurons, such as dopaminergic neurons, from human glial progenitor cells (hGPCs) offers a powerful tool for studying neurological disorders and developing innovative cell- and gene-based therapies. This protocol uses direct reprogramming techniques to efficiently convert human cells into functional neurons. The 3D spheroid model bridges the gap between traditional 2D cultures and *in vivo* models, enhancing physiological relevance. The 3D model accelerates neuronal maturation, as demonstrated by earlier electrophysiological functionality than in 2D cultures, provides complex cell-cell interactions, mimicking *in vivo* architecture, and supports long-term culturing.

The reprogramming process integrates the self-assembly of cells into spheroid structures, without substrate interaction, combined with the lentiviral delivery of transcription factors and shRNAs to induce direct neuronal reprogramming by activation of the factors via doxycycline administration. This streamlined system enables the production of 3D induced dopaminergic neurons (3D iDANs) in the form of spheroids, eliminating the need for specialized equipment. The 3D iDANs generated through this protocol have features of human midbrain dopaminergic neurons, including the expression of dopaminergic and neuronal markers at both mRNA and protein levels. These neurons display functional properties such as action potential firing, synaptic activity, and dopamine release. Furthermore, the 3D environment accelerates neuronal maturation, promotes synaptic connectivity, and supports long-term maintenance.^1^

This protocol marks an advancement in the reprogramming field, providing a robust and scalable approach for generating human subtype-specific neurons. Its versatility allows for adaptation with alternative reprogramming factors to produce other neuronal subtypes, making it highly applicable for research.

### Institutional permissions

All experiments involving human cells were conducted in accordance with relevant institutional and national ethical guidelines and regulations. Researchers intending to use this protocol must obtain the necessary permissions from their own institutions and comply with local biosafety, ethical, and regulatory requirements governing the use of human cells and viral vectors.

## Key resources table

**Table:** 

REAGENT or RESOURCE	SOURCE	IDENTIFIER
**Antibodies**		

DDC rabbit (1:500)	Merck Millipore	Cat# AB1569, RRID:AB_90789
Donkey anti-chicken IgG (H+L) Cy3 (1:200)	Jackson ImmunoResearch	Cat# 703-165-155, RRID:AB_2340363
Donkey anti-mouse IgG (H+L) Alexa 488 (1:200)	Jackson ImmunoResearch	Cat# 715-545-150, RRID:AB_2340846
Donkey anti-rabbit IgG (H+L) Alexa 647 (1:200)	Jackson ImmunoResearch	Cat# 711-605-152, RRID:AB_2492288
Donkey anti-sheep IgG (H+L) Cy3 (1:200)	Jackson ImmunoResearch	Cat# 713-165-147, RRID:AB_2315778
GFAP Chicken (1:1,000)	Merck Millipore	Cat# AB5541, RRID:AB_177521
PDGFRa Rabbit (1:300)	Cell Signaling	Cat# 5241, RRID:AB_10692773
TAU (HT7) Mouse (1:500)	Thermo Fisher Scientific	Cat# MN1000, RRID:AB_2314654
TH Sheep (1:1,000)	Merck Millipore	Cat# AB1542, RRID:AB_90755

**Chemicals, peptides, and recombinant proteins**		

Accutase	Thermo Fisher Scientific	Cat# A1110501
Antibiotic–Antimycotic	Thermo Fisher Scientific	Cat# 15240096
B27 Supplement	Thermo Fisher Scientific	Cat# 12587010
β-Mercaptoethanol	Sigma-Aldrich	Cat# M6250-100mL
Biotin	Sigma-Aldrich	Cat# B4639
Bovine serum albumin (BSA) 7.5%	Thermo Fisher Scientific	Cat# 15260037
CHIR99021	Axon Medchem	Cat# 1386
DAPI	Sigma-Aldrich	Cat# D9542
db-cAMP	Sigma-Aldrich	Cat# D0260
DMEM/F-12, HEPES	Thermo Fisher Scientific	Cat# 11330-032
DMSO	Miltenyi Biotec	Cat# 170-076-303
Donkey serum	Biowest	Cat# S2170
Doxycycline hyclate	Sigma-Aldrich	Cat# D9891-1G
DPBS (no calcium, no magnesium)	Thermo Fisher Scientific	Cat# 14190
Ethanol 70%	Solveco	Cat# 1054
Ethanol absolute	Solveco	Cat# 1015
Hank’s HBSS (no Ca/Mg/phenol red)	Thermo Fisher Scientific	Cat# 14175-046
Hanks HBSS with Phenol Red	Thermo Fisher Scientific	Cat# 14170112
KnockOut Serum Replacement (KSR)	Thermo Fisher Scientific	Cat# 10828010
Laminin	Thermo Fisher Scientific	Cat# 23017-015
LDN-193189	Axon Medchem	Cat# 1509
Liquid nitrogen, N_2_ and cryosystem	CBS cryosystem 4001 value added	N/A
LM-22A4	R&D Systems	Cat# 4607
MEM Non-essential amino acids	Thermo Fisher Scientific	Cat# 11140050
N1 Supplement	Sigma-Aldrich	Cat# N6530
NDiff227	Takara Bio	Cat# Y40002
OCT	Histolab	Cat# 45830
Paraformaldehyde 4% (PFA)	Sigma-Aldrich	CAS 30525-89-4
Penicillin/Streptomycin (10,000 U/mL)	Thermo Fisher Scientific	Cat# 15140122
Poly-L-Ornithine	Sigma-Aldrich	Cat# P4957
Recombinant Human GDNF Protein	R&D Systems	Cat# 212-GD-010
Recombinant Human IGF-I Protein	R&D Systems	Cat# 291-G1-200
Recombinant Human Noggin Protein	Miltenyi Biotec	Cat# 130-103-456
Recombinant Human NT-3 Protein	R&D Systems	Cat# 267-N3-025
Recombinant Human PDGF-AA Protein	R&D Systems	Cat# 221-AA-050
SB-431542	Axon Medchem	Cat# 1661
Sodium azide, NaN_3_	Sigma-Aldrich	S2002
Sucrose	Fisher Scientific	CAS 57-50-1
T3	Sigma-Aldrich	Cat# T5516-1mg
Triton X-100	Thermo Fisher Scientific	Cat# A16046AE
Trypan blue	Thermo Fisher Scientific	T10282
Valproic acid (VPA)	Merck Millipore	Cat# 676380
Virkon	Lanxess	N/A

**Critical commercial assays**		

QIAshredder	QIAGEN	Cat# 79656
RNeasy Mini Kit	QIAGEN	Cat# 74104
RNase-Free DNase Set	QIAGEN	Cat# 79254

**Experimental models: Cell lines**		

RC17 hESCs	Roslin Cells	hPSCreg RCe021-A, RRID:CVCL_L206

**Recombinant DNA**		

FUW-M2rtTA	Addgene	#20342; RRID:Addgene_20342
pLV.tetO.Ascl1.U6.shRest1.U6.shRest2	Addgene	#234846; RRID:Addgene_234846
pLV.tetO.lmx1a	Addgene	#234847; RRID:Addgene_234847
pLV.tetO.Nurr1	Addgene	#234848; RRID:Addgene_234848

**Software and algorithms**		

ImageJ	NIH	v2.3.0/1.53q
LAS X software	Leica	N/A
R	The R Project	v4.2.10/4.1 – https://www.r-project.org/

**Other**		

100 μm Cell strainer	Corning	Cat# 431752
1.5 mL Microcentrifuge tubes	Eppendorf	Cat# 022431021
15 mL Centrifuge tube	Falcon	Cat# 352196
150 mL Bottle top vacuum filter, 0.22 μm	Corning	Cat# 431161
3.5 mL Transfer pipette	Sarstedt	Cat# 86.1171.001
5 mL Serological pipette	Sarstedt	Cat# 86.1253.001
Automated Cell Counter (e.g., Countess)	Thermo Fisher Scientific	N/A
Automated Cell Thawing system (e.g., ThawSTAR)	Biolife Solutions	N/A
Cell Lifter	Fisher Scientific	Cat# 08-100-240
Corning 96-well ULA plate	Corning	Cat# 7007
Costar 6-well TC-treated plate	Corning	Cat# 3516
Cryomolds	Fisher Scientific	Cat# NC9806558
Cryostat	Thermo Fisher Scientific	CryoStar NX70
Cryovial	Sarstedt	Cat# 72.380
Filter paper	Munktell	Cat# 1002
FluorSave Reagent	Merck Millipore	Cat# 345789-20ML
Fume hood	Lab	N/A
Glass slides	VWR	Cat# 631-1551
Laminar Hood Mars Class 2	LaboGene	N/A
Magnetic stirrer	N/A	N/A
Microcentrifuge (2 mL rotor)	VWR	Micro Star 17
NanoDrop	Thermo Fisher Scientific	N/A
Orbital shaker	IKA VIBRAX-VXR	N/A
μ-Plate 24-well Black ID 14 mm	ibidi	Cat# 82426
P1000 wide-bore tips	VWR	Cat# 732-3911
PAP-Pen	Sigma-Aldrich	Cat# Z672548-1EA
Pipette tip 1,000 μL	Sarstedt	Cat# 70.3050.275
Synthetic brush	Slöjd detaljer	Cat# 1423-0000
Superfrost Plus microscope slides	VWR	Cat# 631-0108

## Materials and equipment

**Table:** Preparation and aliquoting of small molecules

Component	Stock concentration	Solvent/ Dilutant
Biotin	100 μg/mL	Add 100 μL of NaOH to 5 mg product, then add 500 μL of Milli-Q water to get a conc. of 10 mg/mL, this is the stock solution. Make 20 μL aliquots and store at −20°C up to 3 months. To prepare a working stock, take a 20 μL aliquot and add 1980 μL Milli-Q water to get a conc. of 100 μg/mL. Make 300 aliquots and store at −20°C up to 3 months. Thaw at 4°C and use within one week.
db-cAMP	50 mM	Dissolve in Milli-Q water and sterile filter through 0.2 μm syringe filter. Make 1 mL aliquots.
CHIR99021	10 mM	Dissolve in DMSO. Make 10 μL aliquots.
Laminin	1 mg/mL	Thaw in fridge on ice. Keep cold, make 50 μL aliquots.
LDN-193189	10 mM	Dissolve in DMSO. Make 10 μL aliquots.
LM-22A4	20 mM	Dissolve in DMSO. Make 10 μL aliquots.
Recombinant human GDNF	20 μg/mL	Add 2.5 mL 0.1% BSA in DPBS (no calcium, no magnesium) to 50 μg product. Make 50 μL aliquots.
Recombinant human IGF	100 μg/mL	Add 2 ml of 0.1% BSA in DPBS (no calcium, no magnesium) to 200 μg product. Make 50 μL aliquots and store at −20°C for 3 months.
Recombinant human Noggin	100 μg/mL	Add 100 μL Milli-Q water and then 900 μL 0.1% BSA in DPBS (no calcium, no magnesium) to 100 μg product. Make 50 μL aliquots.
Recombinant human NT3	10 μg/mL	Add 2.5 mL of 0.1% BSA in DPBS (no calcium, no magnesium) to 25 μg product. Make 50 μL aliquots.
Recombinant human PDGF-AA	100 μg/mL	Add 500 μL of sterile 4 mM HCl to 50 μg product. Make 50 μL aliquots and store at −20°C for 3 months.
SB-431542	20 mM	Dissolve in DMSO. Make 30 μL aliquots.
T3	100 μg/mL	Resuspend 1 mg product in 0.2 mL of 1 M NaOH, then add 9.8 ml of sterilized ddH_2_O to give 100 μg/mL. Make 300 μL aliquots.
Valproic Acid (VPA)	1 M	Dissolve in Milli-Q water and sterile filter through 0.2 μm syringe filter. Make 200 μL aliquots.

All stocks should be prepared before starting. For small molecules diluted to a specific molarity, the required solvent volume must be calculated based on the batch-specific molecular weight of the product with the formula Mass (g) = Concentration (mol/L) x Volume (L) x Molecular Weight (g/mol). Aliquots are stored long term at −20°C for up to 6 months unless otherwise stated. Before use, take out from long term storage and thaw at 4°C, while molecules diluted in DMSO need to be thawed at room temperature (20°C–25°C). Thawed aliquots can be stored at 4°C for 1 month unless otherwise stated.

**CRITICAL:** LDN-193189 is light sensitive. Protect the working stock solution from light during preparation.

### Glial medium

This medium is used for maintaining human glial progenitor cells.

**Table:** 

Component	Stock concentration	Final concentration	Volume for 100 mL
DMEM/F-12, HEPES	N/A	N/A	95.2 mL
B27 Supplement	50X	1X	2 mL
N1 Supplement	100X	1X	1 mL
MEM Non-Essential amino acids	100X	1X	1 mL
Antibiotic-antimycotic	100X	0.5X	0.5 mL
T3	100 μg/μL	60 ng/mL	60 μL
db-cAMP	50 mM	1 μM	2 μL
Biotin	100 μg/mL	100 ng/mL	100 μL
Recombinant human PDGF-AA	100 μg/mL	10 ng/mL	10 μL
Recombinant human IGF-1	100 μg/mL	10 ng/mL	10 μL
Recombinant human NT-3	10 μg/mL	10 ng/mL	100 μL

Combine DMEM/F-12, B27, N1, MEM Non-essential amino acids, antibiotic-antimycotic in a sterile 150 mL filter bottle and sterilize through the 0.22 μm filter. Add T3, db-cAMP, Biotin, PDGFR-AA, IGF-1, NT-3 to the mixture. Store at 4°C for up to one week.

**Table:** Thawing medium

Component	Stock concentration	Final concentration	Volume for 10 mL
DMEM/F-12, HEPES	N/A	N/A	9.5 mL
KnockOut Serum Replacement (KSR)	N/A	5%	0.5 mL

Combine DMEM/F-12 and KSR in a 15 mL centrifuge tube and mix. Prepare fresh.

### NDiff medium

This is the base medium used for Early and Late NDiff medium.

**Table:** 

Component	Stock concentration	Final concentration	Volume for 500 mL
NDiff 227 Medium	N/A	N/A	495 mL
Penicillin/Streptomycin	10,000 U/mL	1%	5 mL

Mix NDiff 227 Medium with Penicillin/Streptomycin, make 40 mL aliquots and store at −20°C for up to 6 months. Thaw aliquot in fridge before use for Early and Late NDiff medium.

**Table:** Doxycycline 50 mg/ml stock

Component	Stock concentration	Final concentration	Volume for 20 mL
Doxycycline hyclate	N/A	50 mg/mL	1 g
DPBS no calcium, no magnesium	N/A	N/A	20 mL

Prepare a 50 mg/mL stock of doxycycline by mixing 1 g with DPBS (no calcium, no magnesium). Make 50–100 μL stocks and store at −20°C for 6–12 months. Before use, take out and thaw at 4°C, and can be stored at 4°C for up to one month.

**CRITICAL:** Doxycycline is light sensitive. Protect the working stock solution from light during preparation.

### Early NDiff medium

This medium is used between D3 and D9 of direct reprogramming.

**Table:** 

Component	Stock concentration	Final concentration	Volume for 40 mL
NDiff medium	N/A	N/A	40 mL
CHIR99021	10 mM	2 μM	8 μL
SB-431542	20 mM	10 μM	20 μL
Recombinant human Noggin	100 μg/mL	50 ng/mL	20 μL
LDN-193189	10 mM	0.5 μM	2 μL
Valproic Acid (VPA)	1 M	1 mM	40 μL
LM-22A4	20 mM	2 μM	4 μL
Recombinant human GDNF	20 μg/mL	2 ng/mL	4 μL
Recominant human NT-3	10 μg/mL	10 ng/mL	40 μL
db-cAMP	50 mM	0.5 mM	400 μL

Store at 4°C for up to one week, add doxycycline fresh before every media change.

### Late NDiff medium

This medium is used between D9 and D21 of direct reprogramming.

**Table:** 

Component	Stock concentration	Final concentration	Volume for 40 mL
NDiff medium	N/A	N/A	40 mL
LM-22A4	20 mM	2 μM	4 μL
Recombinant human GDNF	20 μg/mL	2 ng/mL	4 μL
Recombinant human NT-3	10 μg/mL	10 ng/mL	40 μL
db-cAMP	50 mM	0.5 mM	400 μL

Store at 4°C for up to one month, doxycycline is added fresh to the media prior to every media change.

**Table:** Triton X-100 stock (10%) solution

Component	Stock concentration	Final concentration	Volume for 100 mL
Triton X-100	100%	10%	10 mL
ddH_2_O	N/A	N/A	90 mL

Dissolve using a magnetic stirrer. Store at 4°C. Solution is stable for months.

**CRITICAL:** Triton X-100 is hazardous and needs to be always handled under a fume hood and with protective measures.

**Table:** Antibody blocking solution

Component	Stock concentration	Final concentration	Volume for 10 mL
DPBS no calcium, no magnesium	N/A	N/A	9.4 mL
Triton X-100 stock (10%) solution	10%	0.1%	100 μL
Donkey serum	N/A	5%	500 μL

Prepare fresh.

***Note:*** This table describes the volume required for approximately 5-8 cryosectioned spheroids on a glass slide considering 200-600 μL per slide at each incubation step (i.e., permeabilization, primary antibody, secondary antibody conjugated to fluorescent dyes).

***Alternatives:*** Use serum from the same species in which the secondary antibody was produced (e.g., goat serum for goat anti-rabbit secondary antibodies). Avoid using serum from the same species as the primary antibody (e.g., do not use goat serum with goat primary antibodies).

**Table:** Sucrose 30%

Component	Stock concentration	Final concentration	Volume for 500 mL
Sucrose	N/A	30%	150 g
DPBS no calcium, no magnesium	N/A	N/A	Up to 500 mL

Dissolve using a magnetic stirrer. Store at 4°C. Solution is stable for months.

**Table:** RLT buffer

Component	Stock concentration	Final concentration	Volume for 5 mL
RLT (RNeasy Mini Kit)	N/A	N/A	5 mL
β-mercaptoethanol	14.3 M	1%	50 μL

Prepared solution is stable at room temperature (20°C–25°C) for 1 month.

**CRITICAL:** β-Mercaptoethanol is hazardous and needs to be always handled under a fume hood and with protective measures.

### DNase I stock solution

Dissolve the lyophilized DNase I (1,500 Kunitz units) in 550 μL of RNase-free water (provided by RNase-Free DNase Set) according to manufacturer’s instructions (https://www.qiagen.com/us/resources/resourcedetail?id=b0ca9e5a-ff87-476e-811b-ff80e4f07b3f&lang=en). Aliquot into single-use aliquots, and store at −20°C for up to 9 months.

## Step-by-step method details

### Cell culture plate preparation for glial cell thawing and maintenance

**Timing: 48 h**

1. Preparing tissue culture plates for glial thawing and maintenance
  a. Coat 6-well tissue culture plates with ready-to-use Poly-L-Ornithine (PO) solution (1 mL per well) and incubate the plates at 37°C overnight (approximately 16–20 hours).
  b. Wash the wells three times with DPBS, coat the plates with laminin (5 μg/mL) in HBSS solution with phenol red (1 mL per well) and incubate them at 37°C for 2 hours – 3 days. No washing is needed before use.

***Note:*** We routinely incubate laminin-coated plates in a humidified incubator at 37°C overnight (approximately 16–20 h).

### Thawing and expansion of human glia progenitor cells

**Timing: 1 h (step 2)**

**Timing: 14 days (step 3)**

**Figure 1:**
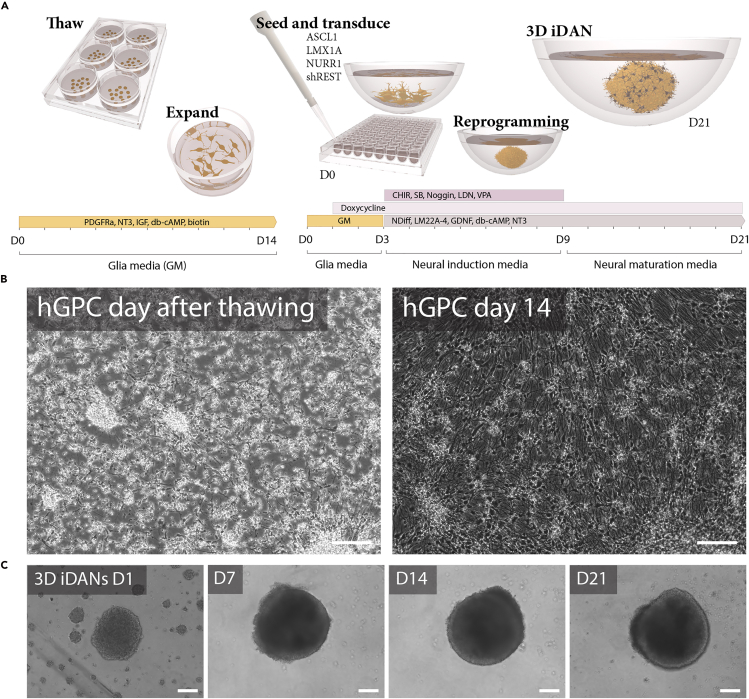
Overview of the 3D reprogramming protocol and representative cell morphology (A) Schematic illustration of the reprogramming protocol workflow showing the key steps: thawing of human glial progenitor cells (hGPCs), seeding and transduction for 3D reprogramming, and progression through media changes (glial medium, early and late NDiff medium). (B) Bright-field images demonstrating representative morphology at key stages: hGPCs one day after thawing, showing healthy adherent cells; hGPCs at 14 days post-thawing, the optimal time point for harvesting and reprogramming. Scale bar: 100 μm. (C) Representative images of 3D-induced dopaminergic neurospheroids (iDANs) at Day 1, 7, 14 and 21 are shown. Scale bar: 100 μm.

This section outlines the steps to thaw and expand one cryovial of hGPCs into two wells of a PO and laminin pre-coated 6-well plate. The goal is to establish healthy cultures (Figure 1B), ensuring they are ready for direct reprogramming. These hGPCs proliferate and migrate out from glial clusters colonizing the surface of the well plate within 14 days.

***Note:*** Detailed protocols describing differentiation of hESCs into hGPCs, including culture conditions, cell identity validation and cryopreservation, are available in Nolbrant et al.^3^ and Wang et al.^4^ Although not the primary focus of this protocol, characterizing the identity of the starting hGPC population ensures reproducible reprogramming outcomes. We perform flow cytometry approximately 12 days post-thaw to assess glial identity using antibodies against CD140a, CD44, and CD133/1.^3^ Immunocytochemistry is also used to confirm the expression of glial markers such as PDGFRA and GFAP. Additional markers that support characterization include OLIG2, SOX10 and NKX2.2.^4^ We recommend proceeding with reprogramming using cell batches that show >40% CD140^+^ cells, <1% SSEA-4^+^ cells and display characteristic glial morphology (Figure 1B). No purification step was performed in this protocol, but CD140^+^ cells can be enriched via FACS if higher stringency is desired.^3^

***Note:*** hGPCs used in this protocol were derived from human embryonic stem cells (hESCs RC17, Roslin Cells, passages 26–30), which were maintained in StemMACS IPS-Brew XF medium on LN521-coated tissue culture plates (0.5 μg/cm^2^). hESCs were passaged weekly using EDTA (0.5 mM). Alternatively, hESCs from other stem cell repositories (e.g., H9 lines) can be used.

2. Thawing glia.
  a. Prepare a 15 mL centrifuge tube containing 10 mL of pre-warmed thawing medium and turn on the automated cell thawing system.
  b. Retrieve a hGPC cryovial from liquid nitrogen storage, and thaw it using the automated cell thawing system, which stops the process while a small ice core remains that will dissolve in the next step.
  c. Add 500 μL of pre-warmed thawing medium dropwise into the cryovial to acclimate the cell suspension and transfer it to the 15 mL centrifuge tube containing thawing medium.
  d. Centrifuge at 300 × g for 6 minutes at room temperature (20°C–25°C).
  e. Carefully aspirate the supernatant and resuspend the cell pellet in 4 mL pre-warmed glial medium.
  f. Seed 2 mL of cell suspension into each well of a PO and laminin pre-coated 6-well plate.
  g. Gently swirl the plate to ensure even distribution and incubate it in a humidified incubator at 37°C and 5% CO_2_.

***Note:*** One confluent well is frozen in one cryovial. Upon thawing, one vial is seeded in 2 wells resulting in a 1 in 2 expansion.

**CRITICAL:** Ensure that cells and cell clusters are plated evenly across the wells to avoid uneven growth.

**CRITICAL:** When retrieving cryovials from liquid nitrogen storage, use proper protective equipment and take all needed safety precautions.

3. Expansion of glia.
  a. Change glial medium (GM) every 2–3 days to support cell metabolic needs and promote proliferation.
  b. Monitor cell confluency daily by visual inspection using a phase-contrast inverted microscope.
    ***Note:*** Representative morphologies and densities are provided in Figure 2A.
    **Figure 2:**
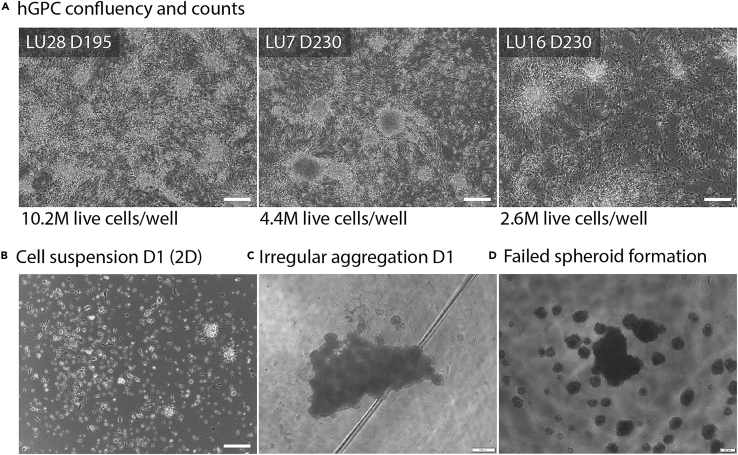
Representative cell morphologies (A) Bright-field images demonstrating representative cell confluency of 3 independent hGPC differentiation batches (LU28 D195; LU7 D230; LU16 D230. Day indicates time of freezing) at 14 days post thawing. Confluency was determined by cell counting. M: Million. Scale bar: 100 μm. (B) An example of hGPCs the day after seeding (D1) on adherent plates following Accutase treatment to show typical single cell suspension with tiny clusters. Scale bar: 100 μm. (C) An example of irregularly shaped spheroid at D1. This shape is often observed and will typically form well defined spheroids after a few days in culture. Scale bar: 200 μm. (D) An example of failed spheroid formation is also included, showing the presence of satellite spheroids and incomplete aggregation. Scale bar: 200 μm.
  c. Cells reach a confluency between 4 × 10^6^ and 8 × 10^6^ cells (depending on the batch) per well within 14 days (Figure 1B).
    **CRITICAL:** To ensure optimal cell viability and reproducibility, it is essential to always use pre-warmed cell medium whenever media comes into contact with cells. Cold medium can cause temperature shock, leading to cellular stress, reduced survival, and impaired reprogramming efficiency. Before each use, pre-warm all media to 37°C in a water bath or incubator to maintain stable culture conditions and support healthy cell growth.

### Spheroid formation and lentiviral transduction (day 0)

**Timing: 1 h**

This step involves the aggregation of cells into 3D spheroids, which serves as the foundation for direct neuronal reprogramming. Lentiviral transduction introduces reprogramming factors into the cells, and doxycycline initiates the process of neuronal induction. Proper spheroid formation and effective transduction are critical for achieving high reprogramming efficiency and consistent outcomes, which is achieved by co-seeding glial cells with lentiviral vectors at the time of spheroid formation to ensure uniform and efficient uptake.

4. Harvest the hGPCs for spheroid aggregation.
  a. Using a sterile cell scraper, gently scrape the hGPCs from the well surface and transfer the harvested cells to a 15 mL centrifuge tube using a 5 mL serological pipette.
  b. Add 1–2 mL pre-warmed DMEM/F-12 to the scraped wells to wash off any remaining cells and transfer it to the same 15 mL tube.
  c. Centrifuge at 300 × g for 6 minutes at room temperature (20°C–25°C).
  d. Discard the supernatant and resuspend the cell pellet in Accutase at room temperature (20°C–25°C). Use a volume approximately equal to the pellet volume (*e.g.*, 100 μL for a 100 μL pellet).
  e. Incubate the tube in the incubator at 37°C for a total of 8 minutes. After 4 minutes, gently pipette the suspension using a P100 to aid dissociation and return to incubation for the remaining 4 minutes.
    **CRITICAL:** This step is critical for cell viability, and Accutase incubation time and pipetting should be adjusted based on how easily the cell clusters dissolve. Carefully monitor cell suspension, and if large clusters are no longer visible, the dissociation is likely sufficient. It is not necessary to achieve complete single-cell suspension as retaining small clusters (≤ 100 μm) improve viability (Figure 2B).
  f. Add around 750–900 μL pre-warmed DMEM/F-12 to reach a final volume of 1 mL and pipette gently but thoroughly to dissociate any remaining clusters.
  g. Use an automated cell counter (recommended) or hemocytometer with Trypan Blue staining to count cells and assess cell viability.
    ***Note:*** Cell viability and cell number is assessed using Trypan Blue method, either using an automated cell counter or a hemocytometer. Dead cells are stained blue by the Trypan Blue, while live cells remain unstained. Cell viability is calculated as the ratio of live cells to the total number of cells (both dead and live).
    **CRITICAL:** Ensure that the viability is above 60% for successful downstream applications.
  h. Centrifuge the cells at 300 × g for 6 minutes at room temperature (20°C–25°C).
  i. Discard the supernatant and resuspend the cell pellet to a density of 100,000 cells per 50 μL in pre-warmed glial medium.
    **Example:** If you have 4 × 10^6^ viable cells, resuspend them into a total of 2 mL of glial medium to achieve the desired concentration of 2 × 10^6^ cells/mL (*i.e.*, 100,000 live cells per 50 μL). After adding the virus suspension (step 7a), the total volume will slightly exceed 2 mL; however dispense 50 μL per well (step 7c). This will allow the formation of approximately 40 spheroids.
    ***Note:*** This protocol provides examples for preparing cell suspensions for spheroid formation and transduction. Adjust cell numbers and volumes as needed to match your experimental design.
5. Prepare lentiviral transduction.
6. Determine the required volume of lentiviral suspension.

**CRITICAL:** Use a MOI of 1 for tetO-*Lmx1a*, tetO*-Nurr1* and MOI of 2 for tetO*-Ascl1*-U6-sh*REST*seq1-U6-sh*REST*seq2 and FUW-*M2rtTA*.

***Note:*** The volume required per lentivirus is calculated using the formula: $Volume\;of\;lentiviral\;suspension\;\left(mL\right)=\frac{\text{MOI}\times \text{Number}\;\text{of}\;\text{cells}}{\text{Virus}\;\text{titer}\left(\frac{\text{TU}}{\text{mL}}\right)}$

***Note:*** TU indicates the number of transducing units. To determine the virus titer for new lentiviral productions, we infect HEK293T cells with serial dilutions of the new virus and a reference virus and quantify via WPRE-targeted qPCR by comparing Ct values of the new virus to the reference virus. The reference virus is a GFP-expressing lentivirus that has been titrated by infecting HEK293T cells with serial dilutions of the virus and quantifying the percentage of GFP-positive cells via flow cytometry. The titer (TU/mL) for the reference is then calculated by taking the number of cells at transduction × % GFP-positive cells × dilution factor × 1,000.

***Note:*** MOI stands for “Multiplicity of infection” and refers to the ratio of virus particles to target cells. For example, a MOI of 1 means that, on average, each cell will be exposed to one viral particle. The optimal MOI depends on several factors including target cell type and virus titer, and must balance efficient transduction with potential toxicity. In our protocol, we use a MOI of 1 or 2 for each lentiviral vector.

***Note:*** A fluorescent reporter or other additional viral vectors can be added to the lentiviral cocktail if needed for downstream applications. We recommend using a MOI of 1.

***Note:*** Appropriate control conditions are included to validate reprogramming outcomes. In our protocol, we routinely use two types of controls. The first is a glia-only control consisting of aggregated 3D hGPCs cultured in standard glial medium without reprogramming factors. The second is a media control consisting of aggregated 3D hGPCs cultured in the same neural differentiation medium (early and late NDiff medium) without reprogramming factors. These controls allow for the assessment of culturing related effects on cell behavior. We do not usually use reporter-only vectors, but such controls could be employed to control viral load effects.

7. Transduce cells.
  a. Add the calculated volumes of each lentivirus carrying the reprogramming factors directly into the tube containing the cell suspension.
  b. Gently mix by pipetting. Avoid formation of bubbles or vortexing as these can mechanically damage the viral particles.
  c. Dispense 50 μL of the cell suspension containing lentiviruses directly into each well of a 96-well ultra-low attachment round-bottom plate, ensuring uniform distribution.
  d. Incubate the plate in a humidified incubator at 37°C and 5% CO_2_. The cells typically self-aggregate into spheroids within 1-2 hours.

**CRITICAL:** Ensure the lentiviruses are titrated between 1 × 10^8^ – 1 × 10^10^ TU/mL to avoid excessive dilution of the cell suspension. The volume of viral suspension added should not exceed 10% of the total medium volume per well to avoid diluting the media.

**CRITICAL:** Always assess cell viability after dissociation using Trypan Blue or fluorescence-based dyes. Adjust dissociation protocols based on the observed viability and cell recovery.

**CRITICAL:** Lentiviral work must be conducted in a biosafety level 2 cabinet. Always wear appropriate personal protective equipment, including double gloves and lab coats. After handling lentivirus pipette tips, tubes, and any disposable materials must be treated with 1% Virkon for 10 min for proper inactivation before disposal. Decontaminate all surfaces with 1% Virkon, followed by 70% ethanol to prevent corrosion of metal surfaces. Dispose of biohazard waste according to institutional guidelines to maintain safety and containment.

### Maintenance of transduced cells during reprogramming (day 1–day 21)

**Timing: 21 days**

Following lentiviral transduction (D0), the maintenance of transduced cells is critical to ensure efficient neuronal conversion. This involves monitoring of spheroid formation, stepwise transition into neural induction media, and regular media changes.

8. The day after transduction (D1) gently top up each well with 100 μL glia medium containing doxycycline (final concentration: 5 μg/mL) to bring the total volume to approximately 150 μL per well.

***Note:*** Doxycycline induces transgene expression, while topping up with fresh media replenishes nutrients and dilutes residual virus without disturbing the spheroids.

**CRITICAL:** Inspect wells under inverted microscope to confirm spheroid formation. If spheroids fail to form, consider troubleshooting adjustments.

9. On day three (D3), completely replace the medium in each well with 200 μL of early NDiff medium.
  a. Continue doxycycline (5 μg/mL) in the medium to maintain transgene expression.

***Note:*** Media changes are performed using an aspirator to carefully remove spent media. To minimize suction pressure and accidental aspiration of the spheroids, we recommend attaching a P100-250 pipette tip to the glass capillary in the aspirator tube.

10. Two-thirds of the medium is then exchanged with early NDiff medium on D5 and D7.
  a. Continue doxycycline (5 μg/mL) in the medium to maintain transgene expression
11. At D9, two-thirds of the medium is exchanged with late NDiff medium to promote neural maturation. This is repeated on D11, D13, D15, D17 and D19.
  a. Continue doxycycline (5 μg/mL) in the medium to maintain transgene expression.

***Note:*** The experiment typically concludes at D21, when spheroids are both collected for RNA extraction and fixed with 4% paraformaldehyde (PFA) for downstream analysis (e.g., gene expression analysis and immunocytochemistry).

***Note:*** If extended culture is required, the protocol can be sustained for at least 50 days with doxycycline withdrawn at D21 to allow further maturation.

**CRITICAL:** For the first 7 days post-transduction, the culture medium is considered to contain residual lentivirus. Proper biosafety precautions must be followed, as described for lentivirus transduction.

**CRITICAL:** Additionally, always pre-warm media to 37°C before use to prevent temperature shock and ensure optimal cell viability.

**CRITICAL:** Add doxycycline fresh to the medium before dispensing it into the wells. Do not store doxycycline diluted in medium, as its stability may be compromised over time. Doxycycline is also light sensitive. Protect the working stock solution from light during preparation.

***Note:*** Expected spheroid aggregation after D1, D7, D14 and D21 can be seen in Figure 1C. More irregular shapes at D1 as shown in Figure 2C can also be expected, and these usually also form well-defined spheroids within days.

### RNA extraction from reprogrammed spheroids for gene expression analysis

**Timing: 30 min (step 12)**

**Timing: 2 h (step 13)**

In this section, we outline the steps for RNA extraction from reprogrammed spheroids to assess transcriptional changes associated with glia-to-neuron conversion. Proceed with gene expression analysis using preferred methods such as RT-qPCR or RNA sequencing to assess expression of target genes and confirm successful reprogramming. Here, we provide suggestion for bulk RNA sequencing.

12. Spheroid collection and lysis.
  a. Prepare RLT buffer by adding β-Mercaptoethanol to a final concentration of 1%.
  b. Transfer 5 to 8 spheroids into a 1.5 mL microcentrifuge tube using a 3.5 mL transfer pipette or P1000 wide-bore tips. Allow the spheroids to sediment and carefully remove the cell culture medium.
  c. Wash once with 500 μL DPBS to remove any residual medium, when removing the DPBS be careful to avoid the spheroids, alternatively let the spheroids sediment before removing the DPBS.
  d. Perform cell lysis by adding 350 μL of RLT buffer with β-Mercaptoethanol per tube and pipetting up and down until the spheroids are fully dissolved.
  e. Snap-freeze the lysate on dry ice for 10 minutes, then transfer the tube to −80°C until RNA extraction.

**Pause point:** RNA lysate can be stored at −80°C for several months (typically up to 6–12 months).

**Alternative option:** If proceeding immediately, continue with RNA extraction without freezing and store on wet ice.

**CRITICAL:** β-Mercaptoethanol is hazardous and needs to be always handled under a fume hood and with protective measures.

13. RNA extraction using column-based kit (*e.g.,* QIAshredder and RNeasy Mini Kit).
  a. Thaw lysate on ice and immediately pass the lysate through cell-lysate homogenizer (QIAshredder) columns according to manufacturer’s instructions in the RNeasy Mini Kit from Qiagen to break up residual debris.
  b. Immediately proceed with RNA extraction using RNeasy Mini Kit from Qiagen according to manufacturer’s instructions (https://www.qiagen.com/us/resources/resourcedetail?id=f646813a-efbb-4672-9ae3-e665b3045b2b&lang=en).
    **CRITICAL:** Perform in-column DNase treatment to remove genomic DNA contamination
  c. Elute RNA in RNase-free water and store at −80°C until analysis.
    **CRITICAL:** Assess RNA concentration and purity using Nanodrop spectrophotometer before proceeding with downstream applications.
    ***Note:*** This typically yields 10–120 ng/μL RNA per sample in 50 μL elution volume. To obtain higher RNA concentrations, reduce the elution volume.
    **Pause point:** Extracted RNA samples can be stored at −80°C for several months (typically up to 6–12 months).
    ***Note:*** Proceed with gene expression analysis using the preferred quantitative methods such as RT-qPCR, or RNA sequencing to assess the expression of target genes and confirm successful reprogramming. Here we provide an outline for bulk RNA sequencing.
14. Prepare cDNA libraries using a suitable kit (*e.g.*, TruSeq RNA Library Preparation Kit v2, RS-122-2001 https://emea.support.illumina.com/content/dam/illumina-support/documents/documentation/chemistry_documentation/samplepreps_truseq/truseqrna/truseq-rna-sample-prep-v2-guide-15026495-f.pdf).
15. Sequence the libraries on an appropriate platform (*e.g.,* Illumina NextSeq or NovaSeq) using paired-end reads (*e.g.*, 2 × 150 bp) and adjust sequencing depth based on experimental needs (*e.g.*, ∼37 million reads per sample using a P3 flow cell).
16. Demultiplex and generate FASTQ files using a standard tool (*e.g.*, bcl2fastq v2.20, Illumina).
17. Quantify transcript expression using a tool such as Salmon^5^ with an index built from a relevant transcriptome model (*e.g.*, Ensembl GRCh38 v.99).
18. Perform quality control using appropriate tools (*e.g.*, RSeQC and MultiQC^6^^,^^7^).
19. Import pseudocounts into a statistical environment (*e.g.*, R v4.1) and summarize to gene level using a suitable package (*e.g.*, tximeta^8^).
20. Identify differentially expressed genes using an appropriate method (*e.g.*, DESeq2^9^).
21. Perform any other required analyses using suitable functions such as Principal Component Analysis (PCA) (*e.g.*, prcomp in R stats), heatmaps (*e.g.*, heatmap.2 in R stats), GO term and pathway enrichment analysis using software like GOseq^10^ and EGSEA.^11^

### Cryosectioning of reprogrammed spheroids for immunocytochemistry

**Timing: 30 min (step 22) and overnight incubation (step 22f)**

**Timing: 4 h (step 23)**

**Timing: 2 h (step 24)**

**Timing: 3 days (step 25)**

**Timing: 30 min (step 26)**

This section outlines the preparation, cryopreservation and cryosectioning of 3D spheroids for subsequent immunocytochemical analysis. Cryosectioning enables high-resolution analysis of protein markers.

***Note:*** Cryosectioning is the primary method described here, but alternative approaches are possible depending on experimental goals. Immunostaining can be performed on intact spheroids and visualized following optical clearing protocols, which preserve 3D structure but often require extended processing times and is less compatible with high-throughput workflows.

22. Fix spheroids.
  a. Transfer spheroids into 1.5 mL centrifuge tubes using a 3 mL transfer pipette or P1000 wide-bore tips, wait for them to sediment and carefully remove the cell culture medium.
    ***Note:*** Multiple spheroids can be collected into the same tube.
  b. Wash once with DPBS to remove residual medium.
  c. Fix spheroids by adding 500 μL of 4% PFA per tube and incubate for 20 minutes at room temperature (20°C–25°C).
  d. Perform three washes with 1 mL of DPBS to remove excess PFA.
    **Pause point:** Fixated and washed spheroids can be stored at 4°C in DPBS for up to a week or in bacteriostatic preservative (0.01% sodium azide in DPBS) for a few weeks.
    **CRITICAL:** Sodium azide is commonly used in antibody storage buffers as a preservative but can interfere with certain antibody conjugations. Therefore, compatibility should be confirmed for each antibody.
    ***Note:*** Antibodies used in this protocol are compatible with spheroids that have been stored in sodium azide containing DPBS.
  e. Remove DPBS and add 1 mL of 30% sucrose to each tube.
  f. Incubate overnight (approximately 16–20 hours) at 4°C on an orbital or rotatory shaker.
23. Embedding in OCT medium and freezing
  a. Replace the sucrose solution in each tube with 1 mL mixture of OCT medium and 30% sucrose in equal volumes.
  b. Incubate for 3 hours at room temperature (20°C–25°C).
  c. Using a P100 pipette tip, first dip the tip in OCT medium to coat it, then gently collect the spheroids by utilizing the viscosity of the OCT medium, allowing the spheroids to adhere to the tip, and carefully transfer them into a cryomold pre-filled with OCT medium.
    **CRITICAL:** Transferring the spheroids using a P100 pipette tip dipped in OCT minimizes carryover of sucrose from the previous step, as sucrose carryover can affect the quality of embedding.
  d. Immediately transfer the cryomold to dry ice and freeze for at least 5 minutes.
  e. Store frozen cryomolds at −80°C until needed.
    **Pause point:** Spheroids embedded in OCT can typically be stored at −80°C for several months up to 1 year.
24. Cryosectioning.
  a. Using a cryostat, section the OCT-embedded spheroids at 20 μm thickness and transfer the cryosections onto Superfrost Plus Microscope glass slides using a fine brush.
  b. Store slides at −20°C until needed.

**Pause point:** Cryosectioned samples can typically be stored at −20°C for several months but are best used within 1–3 months.

25. Immunocytochemistry.
  a. Using a PAP pen, draw a hydrophobic barrier around the spheroid sections to prevent reagent spread.
  b. Wash slides in DPBS for 5 minutes.
    ***Optional:*** If sections detach from the slides during staining, a post-fixation step can help improve adherence. Fix the sections with 4% PFA for 10 min at room temperature (20°C−25°C) and wash three times with DPBS.
  c. Incubate each slide with blocking solution (0.1% triton X-100 and 5% donkey serum in DPBS) for at least 1 hour at room temperature (20°C−25°C). Depending on size of area marked with PAP pen, between 200 μL and 600 μL are needed to adequately cover the section.
  d. Prepare primary antibody solutions.
    i. Dilute primary antibodies in the blocking solution.
  e. Remove blocking solution and add an equivalent volume of primary antibody solution per slide. Incubate overnight (approximately 16 – 20 hours) at 4°C in a humidified chamber to prevent drying.
  f. The next day, wash the slides in DPBS three times for 5 minutes each.
  g. Prepare secondary antibody solutions.
    ii. Dilute secondary antibodies in the blocking solution. DAPI may be included for nuclear staining.
  h. Incubate sections with secondary antibody solution for at least 90 minutes at room temperature (20°C−25°C) in a humidified chamber protected from light.
  i. Wash slides in DPBS three times for 5 minutes each.
  j. Remove DPBS and allow the slides to air dry briefly before mounting them with two to three drops of FluorSave Reagent, then cover with a coverslip.
  k. Let the mounting reagent cure at room temperature (20°C–25°C) overnight (approximately 16−20 hours).
26. Analyze the immunostained sections using a fluorescence inverted or confocal microscope to assess marker expression and neuronal identity.

***Note:*** Keep the secondary antibody solution protected from light.

**CRITICAL:** PFA is hazardous and needs to be always handled under a fume hood and with protective measures.

**CRITICAL:** Do not allow cryosections to fully dry during immunostaining, as it could affect the quality of staining. The goal is to ensure that sections are not visibly wet or pooling with liquid, but not completely dry either.

**CRITICAL:** Ensure the mounting medium has hardened before microscopy to avoid compromising the sample.

## Expected outcomes

This protocol enables the direct reprogramming of human glial cells into induced dopaminergic neurons within a 3D spheroid system (Figure 1A). The expected outcomes include successful spheroid formation, efficient neuronal reprogramming and the gene and protein expression of subtype-specific markers characteristic of midbrain dopaminergic neurons such as tyrosine hydroxylase (TH). TH-expressing cells are typically detectable by immunofluorescence as early as day 7, with an average of 565 ± 76 TH+ cells per spheroid.^1^ This population remains stable throughout culture with consistent count at day 14 and 21, and is sustained up to day 100, indicating persistent identity in long-term 3D cultures.^1^

**Figure 3:**
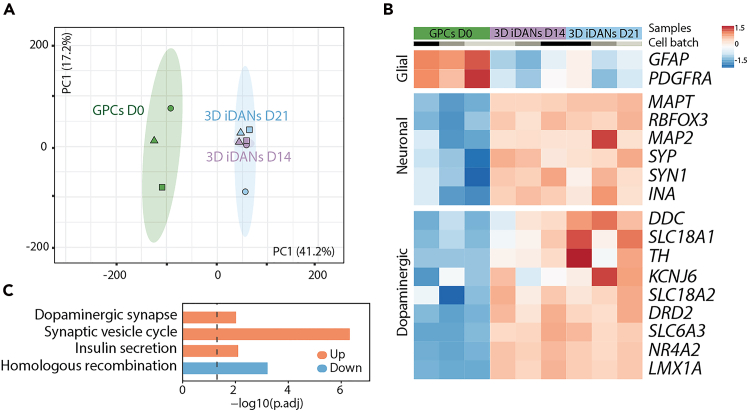
Gene expression validation of 3D dopaminergic reprogramming (A) Principal Component Analysis (PCA): Principal component analysis of whole-transcriptome RNA sequencing data from hESC-derived GPCs at D0 (green), and 3D iDAN spheroids at D14 ( purple) and D21 (blue), with n = 3 biological replicates per time point. Cell batches J1 (circle), J4 (square) and LU28 (triangle). Ellipses represent a 95% confidence interval. (B) Gene Expression Heatmap: Heatmap displaying the expression levels of selected genes (glial, neuronal, and dopaminergic markers) during the 21-day 3D reprogramming of hGPCs into iDANs, analyzed at multiple time points. Values are log-transformed and mean-scaled across all samples. Glial markers (*e.g.*, *PDGFRA* and *GFAP*) are highly expressed at D0 and downregulated over time. Neuronal and synaptic genes (*MAPT, SYN1, SYP, RBFOX3, MAP2,* and *INA*) are upregulated, alongside midbrain dopaminergic markers, indicating successful reprogramming. (C) KEGG Pathway Enrichment Analysis: Bar plot showing the top four KEGG pathways, identified through Enrichment Gene Set Analysis (EGSEA), enriched in 3D iDANs at D21 compared to hGPCs at D0. Bars indicate upregulation (orange) and downregulation (blue), highlighting the significant upregulation of pathways related to “dopaminergic synapse” and “synaptic vesicle cycle” in 3D iDANs.

**Figure 4:**
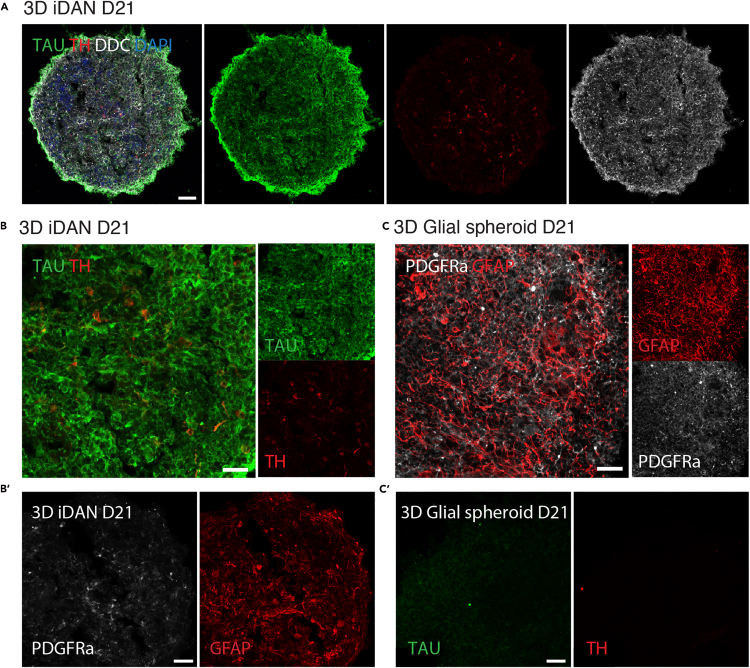
Immunocytochemical characterization of 3D iDANs and glial control spheroids (A) Immunostaining of 3D iDANs at D21 showing expression of neuronal and dopaminergic markers TAU, TH, and DDC, indicating successful reprogramming and neuronal subtype specification. DAPI was used for nuclear staining. Scale bar: 50 μm. (B) Higher magnification of the iDAN spheroid shown in (A), confirming robust expression of neuronal markers and B′) reduced expression of GFAP and PDGFRα . Scale bar: 25 μm. (C) Immunostaining of control glial spheroids (non-reprogrammed) showing strong expression of glial markers GFAP and PDGFRα, and C′) absence of neuronal markers TH and TAU, confirming cell identity prior to reprogramming. Scale bar: 25 μm.

Following the first 24–48 hours post-seeding, spheroids should start to form compact and well-defined aggregates in ultra-low attachment round bottom plates (Figure 1C). Poorly formed or irregular spheroids may indicate suboptimal cell viability or toxicity of the lentiviral cocktail (Figure 2D). Gene expression analysis can be used to assess successful conversion with the downregulation of glial markers and upregulation of neuronal and synaptic genes (Figures 3A–3C). In this 3D reprogramming model, transcriptional reprogramming initiates rapidly following doxycycline induction. A distinct neuronal transcriptional profile is observed within 24 hours of doxycycline addition.^1^ Single-nucleus transcriptomics has revealed that approximately 21% of cells begin expressing neuronal genes as early as day 2, increasing steadily over the first week.^1^ In line with this, by D21, immunocytochemical analysis should reveal the expression of neuronal markers such as TAU, as well as the expression of dopaminergic-specific markers such as TH and DDC (Figures 4A and 4B) and reduction in glial markers such as GFAP and PDGFRa compared to starting glial population (Figures 4B and 4C). Proper adherence to the protocol should result in reproducible and high-quality 3D neuronal cultures suitable for disease modeling, drug screening and regenerative medicine research.

Extended culture beyond day 21 is possible and has been tested for up to 10 weeks with the removal of doxycycline at day 21.^1^ Long-term cultures maintain spheroid integrity and neuronal identity, although marker expression and functional properties continue to evolve over time. For example, we previously observed increased expression of neuronal maturation-associated genes and an increasing proportion of electrophysiologically active neurons over time in culture, indicating ongoing functional and molecular maturation.^1^ This is a major advantage of the 3D system over traditional 2D cultures, as it addresses critical issues related to cell viability, culture integrity and functional stability often compromised in 2D neuronal reprogramming. The 3D environment provides enhanced cell-cell interactions, structural support and long-term maintenance, making it an optimal platform for studies requiring functionality and extended culturing periods. Examples of such studies include electrophysiological recordings to assess neuronal activity, dopamine release assays to assess neurotransmitter functionality and studies of synapse formation using immunostaining for synaptic markers.

## Limitations

While this protocol offers significant advantages for generating functional, subtype-specific dopaminergic neurons suitable for long-term culture, there are inherent limitations associated with the use of 3D culture systems.

Quantitative assessments of reprogramming efficiency, neuronal marker expression, and morphological features are more challenging in 3D systems compared to 2D cultures due to the thickness and opacity of spheroids, which typically require either cryosectioning or optical clearing. Unlike 2D cultures, which facilitate straightforward and scalable image-based analyses, 3D spheroids are less compatible with current automated, high-throughput quantification methods. Cryosectioning of spheroids enables detailed spatial resolution of protein markers but disrupts the overall 3D structure and can truncate cellular morphology, particularly structural processes like neurites. Heterogeneity within spheroids can further lead to variations in cell distribution and marker expression depending on the section analyzed. As a result, a single or few sections may not capture the overall cellular composition. In addition, spheroid density can complicate accurate cell segmentation.

Whole-spheroid imaging remains challenging due to opacity and limited penetration of traditional imaging techniques into the spheroid core, often resulting in incomplete visualization. Optical clearing techniques combined with confocal microscopy provide a solution by enhancing transparency and enabling comprehensive imaging of intact spheroids. We have successfully applied a clearing protocol originally developed for brain slices to our 3D spheroid cultures, confirming its compatibility. However, this requires extended staining, clearing and imaging protocols, and not all antibodies are compatible with clearing agents. For detailed methods on suitable optical clearing techniques, refer to.^12^

For more quantitative analyses, adaptations such as dissociating spheroids into single cells for flow cytometry or performing single-cell transcriptomic can provide higher-resolution data on reprogramming efficiency and cell population characteristics. Detailed workflows for such approaches are described in.^13^ While these techniques address specific limitations, they often involve trade-offs, such as the loss of spatial context in the 3D structure. Additionally, dissociation can introduce mechanical and enzymatic stress to neurons, potentially affecting their survival. It is important to note that we have only tested a dissociation protocol for flow cytometry on glial spheroids and not on reprogrammed neuron spheroids. Therefore, further optimization may be required to ensure the viability and integrity of reprogrammed neuronal populations.

The 3D reprogramming system generates functional iDANs with robust efficiency, though some variability can occur between experiments. Such variation is not unique to the 3D systems and is similarly encountered in 2D cultures, reflecting both biological heterogeneity in the starting hGPC populations and technical factors such as variability in lentiviral co-transduction of all four reprogramming constructs. Despite these limitations, the protocol provides a reproducible system for generating subtype-specific neurons in a relevant 3D context.

## Troubleshooting

### Problem 1

Low viability of hGPCs is observed following dissociation, particularly after Accutase treatment. This may manifest as high cell death or poor recovery of single-cell suspensions, potentially impacting downstream applications such as spheroid formation. Related to step 4.

### Potential solution

Reduce Accutase incubation time and incubate at 37°C for 2 × 2 minutes instead of 2 × 4 minutes. Gently pipette, visually monitor cell suspension in the tube, and stop the process as soon as the solution appears cloudy and clusters have started to dissociate.

Alternatively, skip accutase and mechanically dissociate cells by passing the suspension through a 100 μm cell strainer. This avoids enzymatic damage and can improve cell viability but often leads to higher cell loss because undissociated cell clusters are retained in the strainer.

### Problem 2

Cells fail to self-aggregate or form satellite spheroids after seeding, leading to inconsistent or incomplete 3D structures (related to step 7). This can impact reprogramming efficiency and downstream applications.

### Potential solution

Cell stress or low viability: ensure the viability of the cells is >60% before seeding. Use healthy cultures and avoid prolonged dissociation or handling steps. Technical adjustments: temporarily reduce the seeding volume per well (*e.g.*, to 20–30 μL) for the first 2–4 hours, while keeping cell number constant (*e.g.*, 100,000 cells) to increase cell-cell contact (related to step 4g). Once aggregation begins, top up the media to the recommended volume. Alternatively, centrifuge the plate at low speed (*e.g.,* 200 × g for 5 minutes) to bring cells to the center of the well and promote aggregation (related to step 7d). Begin reprogramming (by adding doxycycline) after the spheroids have formed.

### Problem 3

Spheroids adhere to the inner surfaces of pipette tips during handling (related to step 12 and 22), making it difficult to transfer or manipulate them. This may lead to loss of samples or mechanical damage to the spheroids.

### Potential solution

Coat the pipette tip with 5% bovine serum albumin solution diluted in DPBS before handling spheroids. This creates a protein barrier on the tip surface, preventing spheroids from sticking.

### Problem 4

Significant cell death occurs within 24 hours following virus transduction, leading to a reduced number of viable cells and poor or failed spheroid formation and reprogramming outcomes (related to step 7).

### Potential solution

Reduce the MOI to minimize cytotoxicity while ensuring effective transduction. Begin with a lower MOI and adjust as needed based on the specific cell type and viral titer. If a high virus volume is required due to low titers, consider producing new viral stocks with higher titers to reduce cell stress. Alternatively, shorten the incubation time with the virus, limiting exposure to 6–12 hours instead of overnight (approximately 16 – 20 hours).

### Problem 5

Spheroids are too small or too large for downstream applications, such as imaging, RNA extraction, or electrophysiology.

### Potential solution

The size of the spheroids can be adjusted by modifying the number of seeded cells. Depending on the experimental goal, spheroid size can be tailored by using 10,000 to 500,000 cells per well. Optimize based on the application and downstream requirements.

## Resource availability

### Lead contact

Further information and requests for resources and reagents should be directed to and will be fulfilled by the lead contact, Mette Habekost (mette.habekost@med.lu.se).

### Technical contact

Technical questions on executing this protocol should be directed to and will be answered by the technical contact, Mette Habekost (mette.habekost@med.lu.se).

### Materials availability

• This study did not generate new unique reagents. All materials are purchasable.
• Plasmids used in this study have been deposited to Addgene.

### Data and code availability

• The RNA sequencing data generated in this study have been deposited under accession number GSE295219.
• This study did not generate code.

## Acknowledgments

The authors acknowledge the technical assistance of Jenny Johansson for library preparation and sequencing, Center for Translational Genomics (Lund University) for providing the sequencing service, and Petter Storm for bioinformatic support.

This work was supported by funding to M.H. from Lundbeck Foundation Postdoc Fellowship (R347-2020-2522) and to M.P. from Swedish Research Council (2021-00661), Swedish Parkinson Foundation (Parkinsonfonden), Swedish Brain Foundation, Knut and Alice Wallenberg Stiftelse (KAW 2018-0040), Novo Nordisk A/S, and the Strategic Research Areas at Lund University MultiPark (multidisciplinary research in Parkinson’s disease) and StemTherapy/Lund Stem Cell Center.

## Author contributions

J.G.: conceptualization, investigation, data collection, methodology, and writing – review and editing; K.L.: investigation and writing – review and editing; M.P.: supervision, funding acquisition, writing – review and editing, and visualization; M.H.: conceptualization, investigation, data collection, methodology, formal analysis, writing – original draft, visualization, and supervision. All authors contributed to manuscript review and approved the final version.

## Declaration of interests

M.P. is the owner of Parmar Cells, which holds related intellectual property. M.P. provides paid consultancy for Novo Nordisk and is a member of the scientific advisory board for Arbor Biotechnologies.

## Declaration of generative AI and AI-assisted technologies in the writing process

During the preparation of this work, the authors occasionally used ChatGPT to assist in refining the readability of specific sentences. After using this tool, the authors reviewed and edited the content as needed and take full responsibility for the content of the published article.
